# Levels of hormones regulating appetite and energy homeostasis in response to a 1.5-Year combined lifestyle intervention for obesity

**DOI:** 10.3389/fphys.2023.1010858

**Published:** 2023-02-20

**Authors:** Susanne Kuckuck, Eline S. van der Valk, Anton J. W. Scheurink, Robin Lengton, Mostafa Mohseni, Jenny A. Visser, Anand M. Iyer, Sjoerd A. A. van den Berg, Elisabeth F. C. van Rossum

**Affiliations:** ^1^ Obesity Center CGG, Erasmus MC, University Medical Center Rotterdam, Rotterdam, Netherlands; ^2^ Department of Internal Medicine, Division of Endocrinology, Erasmus MC, University Medical Center Rotterdam, Rotterdam, Netherlands; ^3^ Groningen Institute for Evolutionary Life Sciences, University of Groningen, Groningen, Netherlands; ^4^ Department of Clinical Chemistry, Erasmus MC, University Medical Center Rotterdam, Rotterdam, Netherlands

**Keywords:** appetite regulation, lifestyle intervation, gut hormones, weight regain, gut-brain axis

## Abstract

**Background:** Weight loss can induce changes in appetite-regulating hormone levels, possibly linked to increases in appetite and weight regain. However, hormonal changes vary across interventions. Here, we studied levels of appetite-regulating hormones during a combined lifestyle intervention (CLI: healthy diet, exercise and cognitive behavioral therapy).

**Methods:** We measured levels of long-term adiposity-related hormones (leptin, insulin, high-molecular-weight (HMW) adiponectin) and short-term appetite hormones (PYY, cholecystokinin, gastric-inhibitory polypeptide, pancreatic polypeptide, FGF21, AgRP) in overnight-fasted serum of 39 patients with obesity. Hormone levels were compared between T0 (baseline), T1 (after 10 weeks) and T2 (end of treatment, 1.5 years). T0-T1 hormone changes were correlated with T1-T2 anthropometric changes.

**Results:** Initial weight loss at T1 was maintained at T2 (−5.0%, *p* < 0.001), and accompanied by decreased leptin and insulin levels at T1 and T2 (all *p* < 0.05) compared to T0. Most short-term signals were not affected. Only PP levels were decreased at T2 compared to T0 (*p* < 0.05). Most changes in hormone levels during initial weight loss did not predict subsequent changes in anthropometrics, except for T0-T1 decreases in FGF21 levels and T0-T1 increases in HMW adiponectin levels tended to be associated with larger T1-T2 increases in BMI (*p* < 0.05 and *p* = 0.05, respectively).

**Conclusion:** CLI-induced weight loss was associated with changes in levels of long-term adiposity-related hormones towards healthy levels, but not with orexigenic changes in most short-term appetite signals. Our data indicates that the clinical impact of alterations in appetite-regulating hormones during modest weight loss remains questionable. Future studies should investigate potential associations of weight-loss-induced changes in FGF21 and adiponectin levels with weight regain.

## 1 Introduction

The increasing prevalence of obesity [body-mass-index (BMI) ≥30.0 kg/m^2^] challenges healthcare providers worldwide to counteract the disease (GBD 2015 Obesity Collaborators, 2017; Chooi et al., 2019). When trying to achieve long-lasting treatment success, the mode of weight loss intervention might be essential. In clinical practice, very-low-energy diets are commonly used as they can induce substantial weight loss in a short amount of time (Mustajoki and Pekkarinen, 2001). However, hypocaloric diets are often accompanied by increases in self-reported appetite and followed by weight regain, making long-term weight loss maintenance a critical issue in obesity treatment (Mustajoki and Pekkarinen, 2001; Sumithran et al., 2011). An alternative approach could be to induce a more modest, but sustainable weight loss *via* multidisciplinary treatment.

This commonly observed recidivism in response to diet-induced weight loss has been partly attributed to a physiological compensatory ‘starvation’ response, ranging from a more orexigenic (appetite-inducing) hormonal profile to increased metabolic efficiency favoring energy storage (Sumithran et al., 2011; Sumithran and Proietto, 2013; Greenway, 2015; Lean and Malkova, 2016). Amongst others, this compensatory mechanism may be reflected by normalized levels of long-term adiposity-related hormones (decreases in leptin and insulin levels; increases in adiponectin levels). Although these normalizations indicate metabolic improvements (Haffner et al., 1992; Zhu et al., 2010; Leon-Cabrera et al., 2013), they may favor hunger signaling, decreased energy expenditure and increased energy storage into subcutaneous fat (Kim et al., 2007; Sumithran and Proietto, 2013; Greenway, 2015). Additional contributors may be found in short-term regulators of energy balance which control appetite and metabolism by regulating inter-meal interval (satiety; fasting levels) and meal-termination (satiation; postprandial response) (Lean and Malkova, 2016; Steinert et al., 2017). Important actors in this context include orexigenic hypothalamus-derived agouti-related protein (AgRP), the anorexigenic gut peptides cholecystokinin (CCK), peptide tyrosine-tyrosine (PYY) and pancreatic polypeptide (PP) (Rossi et al., 1998; Sumithran et al., 2011; Sumithran and Proietto, 2013; Lean and Malkova, 2016). In addition, fibroblast growth factor-21 (FGF21) and gastric-inhibitory polypeptide (GIP) are important regulators of glucose metabolism and insulin sensitivity (Seino and Yabe, 2013; Talukdar et al., 2016; Jastreboff et al., 2022).

Normalizations of the long-term adiposity-related hormones leptin, insulin and (HMW) adiponectin levels have been described consistently across different weight loss modalities (Lean and Malkova, 2016; Khosravi-Largani et al., 2019). However, findings are less consistent for short-term regulators of energy balance (Lean and Malkova, 2016). Hypocaloric (very-low-energy) diets seem to induce weight-regain-favoring hormonal alterations (Essah et al., 2010; Sumithran et al., 2011; Greenway, 2015; Lean and Malkova, 2016). In contrast, results from exercise intervention studies rather point towards increased satiety signaling (Lean and Malkova, 2016; Sheikholeslami-Vatani and Rostamzadeh, 2022). A study investigating changes in response to a lifestyle intervention combining a hypocaloric diet and exercise showed no effects on fasting PYY or CCK levels (Coutinho et al., 2018). Thus, weight-loss-induced alterations in fasting levels of appetite-regulating hormones may reflect chronic changes in meal-to-meal appetite regulation which likely vary across interventions and may be relevant in the context of long-term weight-loss-maintenance (Lean and Malkova, 2016).

To our knowledge, no study has yet investigated the changes in levels of appetite-regulating hormones in response to a healthy diet (whereby patients are instructed *not* to follow a low-calorie scheme); combined with exercise and cognitive behavioral therapy (CBT). Moreover, the clinical impact of the proposed weight-loss-induced compensatory mechanism has recently been questioned. Others have noted that previous studies did not clearly demonstrate a link between the shift towards a theoretically orexigenic hormonal disposition and subsequent weight regain (Sumithran et al., 2011; Strohacker et al., 2014; Coutinho et al., 2018; Martins et al., 2020). The aim of this study was to describe changes in serum levels of hormonal long-term and short-term regulators of energy balance during a 1.5-year combined lifestyle intervention (CLI) for obesity which comprises a healthy (‘normocaloric’) diet, exercise and CBT. We also investigated whether changes in hormone levels after initial weight loss may predict subsequent changes in anthropometrics.

## 2 Methods

### 2.1 Participants

The study subjects included adult patients with lifestyle-induced obesity who underwent a multidisciplinary CLI between October 2013 and October 2019. The treatment program was provided by the Obesity Center CGG (‘Centrum Gezond Gewicht’) at Erasmus MC, University Medical Center Rotterdam. Exclusion criteria were other causes of obesity (e.g. genetic or endocrine diseases), inability to speak Dutch, intellectual disability (IQ < 80), current wish for pregnancy and severe physiological or behavioral problems impeding functioning in a group. An inclusion criterion was the presence of ≥1 obesity-related comorbidity (e.g., dyslipidemia, hypertension, non-alcoholic fatty liver disease, type 2 diabetes, obstructive sleep apnea or osteoarthritis). Before starting a trajectory, patients were screened by a medical doctor, a dietician, a physical therapist, and a psychologist. If inclusion criteria were met and no factors necessitating additional treatment were detected, patients were enrolled in the program. All participants provided written informed consent before the start. The study protocol was approved by the local medical ethics committee of the Erasmus MC Rotterdam (MEC 2012–257).

### 2.2 Combined lifestyle intervention (CLI)

Twice a year groups of 10–12 participants started a 1.5-year CLI trajectory. Every group session comprised 1.5 h of combined CBT and nutritional advice which were followed by 1.5 h by exercise sessions. A detailed description is provided in Table 1.

**TABLE 1:** Detailed description of the combined lifestyle intervention (CLI).

Component	Frequency	Duration	Healthcare professional	Details
Combined nutritional advice and cognitive-behavioural therapy (CBT)	• 1x per week in week 1–10	1.5 h	A dietician and a psychologist	The nutrition education protocol focused on promoting a well-balanced healthy diet in accordance with the Dutch food dietary guideline (Voedingscentrum, 2015), whereby participants were instructed *not* to follow a low-calorie diet scheme (therefore, we refer to the diet as ‘normocaloric’). The aim is to help patients implement a healthy diet into their everyday-life which is sustainable on the long-term and will therefore support long-term weight management and health
	• 1x per 2 weeks in week 11–18			
	• 1x per 4 weeks in week 19–26			The psychoeducational sessions were based on CBT and aimed to improve (stress-)coping, impulsivity, self-confidence and usage of the social support system. Techniques that were implemented in these sessions include cognitive restructuring, emotion regulation, increased self-control, problem solving strategies and relapse prevention
	• 1x in 12 weeks in week 26–78			
Exercise session	• 1x per week in week 1–10	1.5 h	Physical therapist	The nutrition and psychoeducation sessions were followed by 1.5 h of exercise. The exercise sessions comprised both aerobic endurance training as well as anaerobic resistance training. The aim was to stimulate exercise in the home-setting and to improve cardiorespiratory fitness and muscle strength. Therefore, the content of exercise sessions varied: In some sessions, patients were subjected to strength and/or endurance training which they could repeat at home, other sessions focused on introducing new sports such as e.g. tennis or basketball in order to stimulate the patients’ exploration of their individual ways to create a more active lifestyle. Moreover, patients received advice on a healthy pattern of physical activity: including 30 min of mild physical activity per day (e.g. walking), as well as 1–2 times endurance training per week (e.g. running), and 1–2 times per week resistance training (e.g. muscle strengthening with weights)
	• 1x per 2 weeks in week 11–18			
	• 1x per 4 weeks in week 19–26			
	• 1x in 12 weeks in week 26–78			
Homework			Dietician, psychologist and physical therapist	During group sessions, homework was given in order to promote the patients’ active participation in the program and to support their own exploration of ways to establish a healthier lifestyle in a personalized way
Medical evaluations	• Baseline		Medical doctors	Patients underwent medical consults in order to changes in relevant clinical parameters (e.g. weight, waist circumference, blood glucose, lipid levels … ), to consult regarding medication use and to take additional blood samples for research purposes
	• After 10 weeks			
	• After 78 weeks (end of program)			

Evaluation moments were at baseline (before the start of the program, T0), after the first 10 weeks of (intensive) treatment (T1) and at 1.5 years at the end of the program (T2). At each time point, anthropometrics and metabolic parameters were assessed for clinical care and additional fasting blood samples were drawn for research purposes. The present analysis represents a retrospective analysis of all available follow-up serum samples in March 2020.

### 2.3 Hormone measurements

For 45 patients, blood serum samples were available at follow-up. The samples were stored after centrifugation at −20 or −80°C for a maximum duration of 7 years. Commercially available enzyme-linked immunosorbent assay (ELISA) kits were used to determine levels of leptin (Mediagnost, Reutlingen, Germany), total PYY, PP, GIP, FGF21 (Millipore Corporation, Billerica, MA, United States) as well as AgRP and CCK (Sigma Aldrich, St. Louis, MO, United States). HMW adiponectin was determined using a chemiluminescent enzyme immunoassay (CLEIA, Lumipulse G1200, Fujirebio, Gent, Belgium). All analyses for leptin, PYY, GIP, FGF21, AgRP, CCK and HMW adiponectin were performed at the same time at the same laboratory at the end of the study. For each patient, samples from all time points were analyzed in the same plate to prevent inter-assay bias. Insulin was measured as part of standard clinical care using a commercially available CLEIA kit for *in vitro* diagnostics immediately after blood drawing (Lumipulse G1200, Fujirebio, Gent, Belgium). Part of this insulin data has been reported in previous articles of our group (e.g. (van der Zalm et al., 2020).

### 2.4 Statistical analysis

All statistical analyses were performed using SPSS version 24 (IBM Corp. 2019). If hormone levels were below the detectable range of the ELISA analyses, missing values were replaced by the limit of detection stated in the protocol. This occurred for PYY (one sample at T2), and PP (two samples at T0, three samples at T1, three samples at T2). To detect within-subject changes across the three time points, repeated-measures ANOVAs with Bonferroni-corrected *post hoc* t-tests were used or Friedman’s test with Bonferroni-corrected *post hoc* Wilcoxon-signed rank tests in case of non-normality. Data is reported as means ± standard error (SEM) or, if not normally distributed, median and interquartile range (IQR). Chi-squared test of independence was used to test for sex differences in proportions of responder status at T1 and T2. Percentage changes of all outcomes were computed using the following formula: $\%\Delta variable\;X\;between\;T0\;and\;T2=\frac{variableX\_T2-variableX\_T0}{variableX\_T0}$ . To detect associations between % changes of parameters, univariable linear regressions were used. Corrections for the potential confounders sex and age were performed using multiple linear regressions. For insulin and HOMA-IR, we performed sensitivity analyses excluding 5 patients who had taken anti-diabetic drugs at any time point. Since results did not notably change after exclusions, we report the results for the full group. Statistical significance was set at *p* < 0.05. For regression analyses, we additionally applied a Bonferroni-correction for multiple testing, resulting in a significance level of $\alpha =\frac{0.05}{9}=0.0055$ .

## 3 Results

### 3.1 Baseline sample characteristics

Of the 45 patients for whom follow-up blood samples were available, six were excluded: two patients were not fasting at blood drawing, one patient became pregnant, one was later diagnosed to have a genetic form of obesity, one patient underwent bariatric surgery and one developed a severe recurring pancreatitis. Thus, we included a total of 39 patients (31 women, 79.5%) who had a mean BMI of 40.6 kg/m^2^ (±0.9 SEM) and a mean age of 41.8 years (±2.1 SEM). Details are provided in Table 2.

**TABLE 2:** Changes in anthropometrics in response to CLI.

	Baseline		10 weeks	1.5 years
	**N**	n (%)/Mean (±SEM)/Median (IQR)	n (%)/Mean (±SEM)/Median (IQR)	n (%)/Mean (±SEM)/Median (IQR)
Female (%)	39	31 (79.5%)		
Age (years)	39	41.8 (±2.1)		
Type 2 Diabetes (%)	39	9 (23.1%)	8 (20.5%)	8 (20.5%)
BMI (kg/m^2^)	39	40.6 (±0.9)	38.5 (±0.9)***	38.5 (±0.9)***
WC (cm)	39	115.4 (±2.4)	108.6 (±2.4)***	109.3 (±2.6)***
Weight (kg)	39	118.6 (±3.1)	112.6 (±2.9)***	112.6 (±3.1)***
Triglycerides (mmol/l)	39	1.27 (0.89–1.76)	1.02 (0.88–1.55)	1.10 (0.82–1.52)
HDL	39	1.22 (1.02–1.51)	1.18 (0.96–1.37)*	1.33 (1.18–1.45)^$$$^
LDL	39	3.24 (±0.15)	2.98 (±0.13)*	3.14 (±0.16)
Hba1c (mmol/l)	39	38 (±0.6)	37 (±0.6)**	37 (±0.6)*
Blood glucose (mmol/l)	39	5.6 (5.2–6.5)	5.2 (4.9–5.9)*	5.5 (5.1–6.2) ^$^
HOMA-IR	37	5.03 (3.23–7.90)	4.19 (2.55–5.16)**	4.32 (2.52–5.84)*

BMI = body-mass-index, WC = waist circumference; HDL = high-density lipoprotein; LDL = low-density lipoprotein; HOMA-IR = Homeostatic Model Assessment for Insulin Resistance. ***p < .001, **p < .01, *p < .05 indicates significant difference compared to baseline; ^$ $ $^ *p* < .001, ^$^ *p* < .05 indicates significant difference compared to 10 weeks. Tested using Bonferroni-corrected repeated-measurements ANOVA.

### 3.2 Changes in anthropometrics in response to CLI

After 10 weeks (T1), patients showed a mean weight loss of −6.1 kg (5.1%) which was maintained after 1.5 years of treatment (T2, -6.0 kg; −5.0%). This was accompanied bya mean decrease in waist circumference (WC) of −6.8 cm (−5.8%) at T1 and -6.1 cm (−5.2%) at T2 (all *p* < 0.001). In addition, we saw improvements in the patients’ blood lipid profile (see Table 2). At T1, 19 patients (48.7%) lost ≥5% weight (‘responder’, of whom 16 were women) and at T2 there were 20 (51.3%) responders (of whom 17 were women). There was no sex effect on responder status at T1 (*p* = 0.378) or T2 (*p* = 0.317).

### 3.3 Changes in levels of appetite-regulating hormones in response to CLI

**FIGURE 1:**
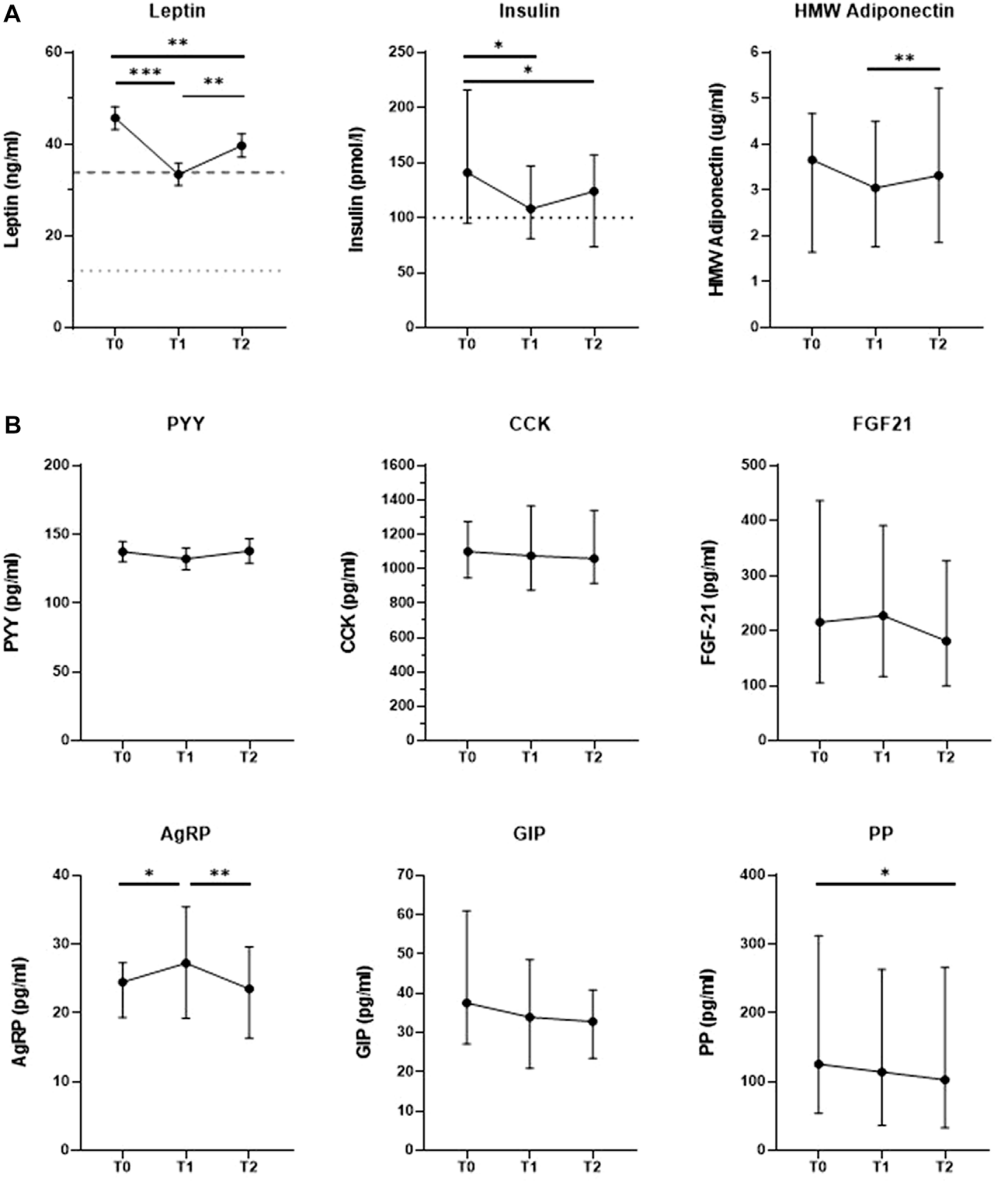
Changes in in levels of hormones regulating appetite and energy homeostasis in response to a combined lifestyle intervention from baseline (T)) to 10 weeks (T1) and 1.5 years of treatment (T2). **(A)** *Changes in levels of long-term adiposity-related hormones, including decreases in leptin and insulin levels towards normal levels. Cut-off lines: Upper limit of reference range for healthy populations (dotted lower line: males: 0.2–12.4 ng/ml; dashed upper line: females: 1.5–33.9 ng/ml), as used for clinical diagnostics in the Erasmus MC. Cut-off line insulin: Upper limit of reference range for healthy populations (≤ 100 pmol/l for both sexes) as used for clinical diagnostics in the Erasmus MC. N-37–39. Data are expressed as mean*+*/*-*SD SEM* (*leptin*) *or median (IQR) (insulin, HMW adiponectin), depending on normal distribution. HMW Adiponectin = High molecular weight adiponectin./.* **(B)** No change in levels of most short-term regulators of energy balance at the end of CLI. PYY = Peptide tyrosine-tyrosine-tyrosine, CCK = Cholecystokinin, FGF21 = Fibroblast growth factor 21, AgRP = Agouti-related protein, GIP = Gastric-inhibitory polypeptide, PP = Pancreatic polypeptide. Data are expressed as mean+/-SD SEM (PYY) or median (IQR) (PP, AgRP, GIP, PYY, CCK, FGF-21), depending on normal distribution. *p < .05, **p < .01, ***p < .001 after repeated-measures ANOVA with Bonferroni-corrected post hoc test or Fridman’s test with Bonferroni-corrected post hoc tests in case of non-normality. N = 38–39.

We investigated changes in hormone levels across the three measurement time points. In Figure 1A, we show that levels of the long-term adiposity-related hormones leptin and insulin were decreased at T1 (n = 39, 33.4 ng/ml ±2.4 SEM, *p* < 0.001; n = 37, 108 pmol/l [81–147 IQR], *p* = 0.011, respectively) as well as at T2 (39.7 ng/ml ± 2.6 SEM, *p* = 0.009; 124 pmol/l [74–157 IQR], *p* = 0.013, respectively) compared to baseline (45.8 ng/ml ± 2.5 SEM, 141 pmol/l [95–216 IQR], respectively). Leptin levels significantly increased between T1 and T2 (*p* = 0.004), which was not observed for insulin levels (*p* > 0.05). At all three time points, leptin and insulin levels of many patients remained above the normal reference ranges used for clinical diagnostics at the Erasmus MC. Specifically, hyperleptinemia (1.5–33.9 ng/ml for women, 0.2–12.9 ng/ml for men) was observed in 34 patients (87.2%) at T0, in 21 patients (53.8%) at T1 and in 27 patients (69.2%) at T2. Similarly, a total of 23 patients (62.2%) had hyperinsulinemia (>100 pmol/l) at T0, 18 (48.6%) at T1 and 19 patients (51.4%) at T2. HMW adiponectin levels did not change significantly between T0 and T1 (n = 39, 3.66 μg/ml [1.65–4.67 IQR] vs 3.05 μg/ml [1.77–4.50 IQR], respectively), but were increased at T2 (3.32 μg/ml [1.86–5.23 IQR]) compared to T1 (*p* = 0.003).

As depicted in Figure 1B, levels of most short-term regulators of energy balance did not change significantly in response to the intervention except for PP and AgRP. PP levels were significantly decreased at T2 (n = 38, 102.75 pg/ml [32.90–265.95 IQR]) compared to T0 (125.65 pg/ml [54.28–312.33 IQR], *p* = 0.049). AgRP levels increased significantly between T0 (n = 38, 24.50 pg/ml [19.33–27.29 IQR]) and T1 (27.25 pg/ml [19.24–35.43 IQR], *p* = 0.009), but decreased again between T1 and T2 (23.44 pg/ml [16.76–30.66 IQR], *p* = 0.012) so that AgRP levels did not significantly differ from baseline after 1.5 years of treatment (*p* < 0.05). We did not find significant between-time-point-differences for PYY, GIP, CCK or FGF21 levels (all *p* > 0.05).

Changes in anthropometrics were associated with changes in leptin, insulin and HMW adiponectin at the end of the program (see Supplementary Table S1). A sensitivity analysis including only women showed results closely paralleling those of the full group (see Supplementary Figure S2).

### 3.4 Associations of initial hormone changes (T0-T1) with subsequent changes in anthropometrics (T1-T2)

Table 3 shows the associations of initial hormone changes (%∆ T0-T1) with subsequent changes in BMI and WC (%∆ T1-T2) before and after correction for sex and age. In the crude analysis, initial decreases in insulin, FGF21 as well as PYY levels were associated with subsequent increases in BMI (*p* < 0.05). Initial increases in HMW adiponectin levels tended to be associated with subsequent increases in BMI, although this association did not reach statistical significance (*p* = 0.070). We did not see significant associations between initial hormone level changes and subsequent changes in WC.

**TABLE 3:** Associations between initial (T0-T1) % changes in hormone levels and subsequent (T1-T2) % changes in anthropometrics.

		Model 1 unadjusted^a)^			Model 2 adjusted for age and sex^b)^		
	***N***	***β (95% CI***)	***Standardized β***	***p***	***β (95% CI***)	***Standardized β***	***p***
Dependent variable: T1-T2 ∆ % **BMI**							
T0-T1 ∆ % Leptin	39	0.004 (−0.075; 0.083)	.017	.917	0.011 (−0.068; 0.090)	.046	.782
T0-T1 ∆ % Insulin	39	**−0.062 (-0.123; -0.001)**	**−.319**	.048*	−0.053 (−0.116; 0.011)	−.271	.102
T0-T1 ∆ % HMW Adiponectin	39	0.097 (−0.008; 0.203)	.293	.070	0.110 (0.000; 0.220)	**.**332	**.**050
T0-T1 ∆ % GIP	38	−0.006 (−0.032; 0.021)	−.070	.675	0.000 (−0.028; 0.029)	.006	.974
T0-T1 ∆ % PP	38	−0.032 (−0.090; 0.026)	−.183	.272	−0.034 (−0.092; 0.023)	−.195	.237
T0-T1 ∆ % PYY	38	**−0.035 (-0.069; -0.002)**	**−.337**	.039*	−0.032 (−0.068; 0.003)	−.308	.074
T0-T1 ∆ % CCK	39	0.035 (−0.037; 0.106)	.159	.333	0.014 (−0.064; 0.092)	.064	.720
T0-T1 ∆ % FGF21	38	**−0.021 (-0.040; -0.003)**	**−.360**	.027*	**−0.021 (-0.040; -0.001)**	−.**350**	.038*
T0-T1 ∆ % AgRP	38	0.040 (−0.025; 0.105)	.203	.221	0.023 (−0.050; 0.096)	.115	.533
Dependent variable: T1-T2 ∆ % **WC**							
T0-T1 ∆ % Leptin	39	−0.055 (−0.144; 0.033)	−.205	.212	−0.047 (−0.135; 0.042)	−.173	.290
T0-T1 ∆ % Insulin	39	−0.046 (−0.118; 0.027)	−.206	.209	−0.033 (−0.107; 0.042)	−.148	.379
T0-T1 ∆ % HMW Adiponectin	39	0.079 (−0.044; 0.202)	.209	.201	0.103 (−0.024; −0.231)	.274	.108
T0-T1 ∆ % GIP	38	−0.018 (−0.047; 0.011)	−.207	.213	−0.015 (−0.045; 0.015)	−.169	.329
T0-T1 ∆ % PP	38	−0.024 (−0.088; 0.040)	−.126	.449	−0.027 (−0.091; 0.036)	−.142	.391
T0-T1 ∆ % PYY	38	−0.027 (−0.066; 0.013)	−.224	.177	−0.019 (−0.061; 0.023)	−.161	.359
T0-T1 ∆ % CCK	39	−0.009 (−0.091; 0.074)	−.035	.833	−0.041 (−0.129; 0.047)	−.164	.353
T0-T1 ∆ % FGF21	38	−0.015 (−0.037; 0.007)	−.220	.184	−0.012 (−0.035; 0.011)	−.175	.309
T0-T1 ∆ % AgRP	38	0.026 (−0.049; 0.101)	.118	.481	0.001 (−0.082; 0.085)	.006	.973

CI = confidence interval; BMI = body-mass-index, WC = waist circumference; HMW, adiponectin = high-molecular-weight adiponectin, GIP = gastric inhibitory polypeptide; PP = pancreatic polypeptide; PYY = peptide tyrosine-tyrosine; CCK = cholecystokinin, FGF21 = fibroblast growth factor 21, AgRP = agouti-related protein. ^a)^ Univariable linear regressions were used with T0-T1% changes in hormone levels as predictor and T0-T2% changes in BMI/WC, as outcomes. ^**b)**^ Adjustment was performed using multiple linear regression.* indicates *p* < .05 (highlighted in bold text). None of the association was significant at the Bonferroni-corrected significance level α = 0.005.95% CI = 95% Confidence interval.

After correction for sex and age, only the association of T0-T1% decrease in FGF21 levels with T1-T2% increase in BMI remained significant (*p* < 0.05). T0-T1% increase in HMW adiponectin was in trend associated with subsequently higher T1-T2% increase in BMI (*p* = 0.05). No other associations were observed between initial (T0-T1) % change in hormone levels and subsequent (T1-T2) % change in BMI or WC (see Table 3). Results did not change notably after an additional sensitivity analysis, correcting for T0-T1%∆ in BMI or WC respectively (see Supplementary Table S3). Neither did the results change notably after a sensitivity analysis including only women (see Supplementary Table S4). When applying the Bonferroni-corrected significance level of *p* < 0.0055, none of the associations remained statistically significant.

## 4 Discussion

Here, we describe changes in fasting levels of long-term and short-term regulators of energy homeostasis in response to a CLI combining a healthy normocalorc diet, exercise and cognitive behavioral therapy. We also addressed the question whether changes in hormonal regulators of energy balance during initial weight loss (T0-T1) predict subsequent changes in anthropometrics (T1-T2).

We found that the modest CLI-induced weight loss (≈5%) (Jensen et al., 2014) was accompanied by changes in levels of long-term adiposity-related hormones (leptin, insulin and HMW adiponectin) towards normal values. Importantly, after 1.5 years, levels of most short-term regulators of energy homeostasis were not altered, except for a decrease in fasting PP levels. When corrected for sex and age, most hormonal changes during initial weight loss did not predict subsequent changes in anthropometrics. Only initial decreases in FGF21 levels and increases in HMW adiponectin levels tended to be associated with larger subsequent increases in BMI.

The observed decrease in leptin and insulin levels, along with the (small) increase in HMW adiponectin levels are likely beneficial for metabolic health (Haffner et al., 1992; Kim et al., 2007; Zhu et al., 2010; Leon-Cabrera et al., 2013). Reductions in anorexigenic leptin and insulin levels may be interpreted as potentially hunger-inducing (Sumithran et al., 2011; Sumithran and Proietto, 2013). However, they may also indicate reduced leptin and insulin resistance, both of which are reflected by high peripheral hormone levels and are associated with metabolic dysfunction as well as disrupted brain appetite signaling (Matheny et al., 2011; Könner and Brüning, 2012; Heni et al., 2014). Consequently, the observed reductions may reflect improved central hormone sensitivity, and thus improved long-term appetite regulation. Indeed, we did see improved whole-body insulin sensitivity at the end of the program, as indicated by decreased HOMA-IR (see Table 1). At any rate, neither initial changes in leptin nor insulin predicted weight regain in the corrected model, suggesting that their influence on long-term weight management was, if present, very limited. It must be noted that throughout the whole intervention, levels of leptin and insulin remained above the normal reference ranges in many of the patients. This is likely due to the fact that, despite weight loss, these patients still had obesity.

At the end of the program, levels of most short-term regulators of energy homeostasis (PYY, CCK, GIP, AgRP and FGF21) were not significantly different from baseline, indicating that there were no major long-term alterations towards a potentially weight regain-inducing disposition. These results partly oppose findings regarding hypocaloric diets, most prominently including decreased PYY and CCK levels (Essah et al., 2010; Sumithran et al., 2011; Sumithran et al., 2013). However, our results are in line with previous studies regarding interventions comprising either increased physical activity/exercise or combinations of exercise and dietary advice (but excluding very-low-energy diets). These studies have reported unaltered levels or even increased PYY and CCK levels as well as decreased GIP levels (Jones et al., 2009; Vos et al., 2011; Guelfi et al., 2013; Martins et al., 2013; Lean and Malkova, 2016; Sheikholeslami-Vatani and Rostamzadeh, 2022). Here, it must be noted that weight loss in these studies was, as in our study, modest or not apparent and results are not completely consistent (Lien et al., 2009; Lean and Malkova, 2016; Ataeinosrat et al., 2022; Sheikholeslami-Vatani and Rostamzadeh, 2022). Thus, whether this (potentially beneficial) lack of strong hormonal alterations is due to the mode or the amount of weight loss/fat mass decrease, we cannot determine here.

A similar line of thought may be followed regarding the lack of changes in FGF21 levels in our study. FGF21 levels have been observed to be increased in obesity and to decrease after ≥9% diet- or surgery-induced weight loss (Christodoulides et al., 2009; Gómez-Ambrosi et al., 2017). This indicates that obesity-related increases in FGF21 levels may represent a compensatory mechanism for disease-related metabolic challenges and weight-loss-induced reductions might actually reflect improved metabolic health. In response to our intervention, FGF21 levels were not significantly altered, which is in line with previous observations in the context of modest weight loss (5.3%) (Mai et al., 2011). Thus, a more pronounced weight loss may be needed to significantly affect FGF21 levels.

The observed decrease in PP levels in our study may, meanwhile, point towards more hunger signaling. Varying effects of weight loss on fasting PP levels have been reported before, ranging from a decrease to an increase to the absence of changes (Reinehr et al., 2006; Sloth et al., 2008; Sumithran et al., 2011; Belinova et al., 2017). This variation in response may be due to differences in the interventions, amount of weight loss, study duration or sample composition. Thus, the effect of weight loss on PP secretion and the implications for weight management remain a subject of further investigation.

Even though we observed an increase in AgRP levels after 10 weeks of initial weight loss, levels had reverted to baseline values at the end of the program. This finding is partly in line with previous studies reporting increased levels of AgRP in response to diet- or lifestyle-induced weight reductions (Patkar et al., 2019; Kravchychyn et al., 2021). It also points towards increased hunger signaling in line with a potential weight-loss-induced physiological compensation (Sumithran et al., 2011; Sumithran and Proietto, 2013; Greenway, 2015). However, the reversal of AgRP levels back to baseline levels at the end of treatment also suggests that a potential acute ‘starvation’ response *via* increased AgRP levels can be silenced once energy balance is maintained on the long-term, i.e. once the body has had time to adapt to a new ‘set point’ of energy stores. Notably, there were no associations between initial increase in AgRP levels and subsequent changes in anthropometrics, indicating that a potential limiting effect on successful weight management was, if present, rather small.

In fact, after corrections for sex and age, most changes in hormone levels during initial weight loss (T0-T1) were not associated with subsequent changes in anthropometrics. Only initial decreases in FGF21 levels and increases in HMW adiponectin levels tended to be associated with subsequent increases in BMI, but not WC.

At first sight, this result seems puzzling since both a decrease in FGF21 levels as well as an increase in HMW adiponectin could indicate metabolic improvements in the context of obesity (Kim et al., 2007; Lee and Shao, 2014; Stern et al., 2016; Gómez-Ambrosi et al., 2017). Data from rodents suggests that both hormones act *via* a shared axis to exert their beneficial lipid- and insulin-lowering effects *via* actions on the adipose tissue and the liver (Holland et al., 2013; Stern et al., 2016). Indeed, FGF21 has been identified as an upstream effector of adiponectin (Holland et al., 2013). Nevertheless, their interaction is likely to be altered in the present context since, during weight loss, people with overweight or obesity usually decrease in their FGF21 levels while simultaneously increasing in adiponectin levels (Soni et al., 2011; Gómez-Ambrosi et al., 2017). Our findings are in agreement with studies suggesting that; in the context of obesity; weight-loss-induced metabolic and hormonal improvements such as increases in adiponectin levels can come at the expense of increased metabolic efficiency and increased energy storage into (especially subcutaneous) fat cells (Kim et al., 2007; Lee and Shao, 2014; Stern et al., 2016). Partly in line with this hypothesis, data from the Nurses’ Health study showed that higher baseline adiponectin levels predicted more subsequent weight gain in a large sample of healthy women (Hivert et al., 2011). Based on a study in rodents, the authors hypothesized that this association may reflect a state of adiponectin-induced improved metabolic efficiency and healthier adipocyte functioning *via* the expansion of subcutaneous body fat (instead of ectopic fat accumulation as e.g. in the pancreas or the liver) (Kim et al., 2007; Hivert et al., 2011). Since we do not have data on the size or functionality of our patients’ adipocytes, a deeper investigation of this hypothesis is unfortunately beyond the scope of this study.

At any rate, our results regarding the association between T0-T1 change in FGF21/HMW adiponectin levels and subsequent T1-T2 increase in BMI must be interpreted with caution. First, because of limited robustness: for both FGF21 as well as HMW adiponectin, the exclusion of the most extreme data points resulted in a non-significant association (see Supplementary Figure S3), indicating that the associations may have been driven by these two data points. Nevertheless, there was no other reason to exclude them, so we kept them in the dataset. Second, as we performed many statistical tests, there is a possibility that the observed association has occurred by chance. Indeed, the association did not remain statistically significant when applying the (however rather conservative) Bonferroni-correction for multiple testing. Finally, the unstandardized regression coefficient of changes in HMW adiponectin levels as a predictor was very small, indicating that for every 1% increase in T0-T1 HMW adiponectin levels, there was a 0.11% increase in BMI between T1 and T2 (see Table 3). The regression coefficient for T0-T1% changes in FGF21 levels was even smaller (see Table 3), indicating that the actual impact of initial hormone changes on subsequent weight gain might not be of strong clinical relevance. Thus, the role of (changes in) FGF21 and HMW adiponectin levels and weight management warrants further investigation in a larger sample.

### 4.1 Strengths

A clear strength of this study is the >1-year follow-up period after initial 10-week period of weight loss, which allowed us to investigate effects of hormonal alterations on long-term weight loss maintenance. Second, we measured a large number of appetite-regulating hormones, which provides a more comprehensive picture of the changes in the homeostatic appetite system than measuring few or single hormones alone.

### 4.2 Limitations

We do acknowledge that this study also has some limitations. First, our results are limited to fasting hormone levels. Although this provides insight into the potential chronic effects of our intervention on long-term satiety (meal-to-meal interval), it would also be of interest to investigate the postprandial hormone release to assess the effects on short-term satiation (postprandial response) as both may affect food intake and long-term weight management in different ways. Second, the current study is limited to the demonstration of associations and does not provide evidence of causality. Third, although patients were instructed to follow a healthy normocaloric diet, we were not able to exactly determine to what degree patients had complied with the intervention regime. Finally, the limited sample size of our study may have resulted in to type 1 errors and our results should thus be replicated in a larger cohort.

### 4.3 Future directions

The limited sample size of the current study prevented us from investigating potential synergistic effects of changes in multiple hormones in a single statistical model. This could be included in future large-scale studies. It would also be highly interesting to prospectively compare the hormonal alterations in response to a normocaloric CLI to a control group and other interventions as well as single components of the intervention (e.g., CLI vs. very-low energy diets; vs. healthy diet only; vs. CBT only; vs. exercise only). Finally, future studies should investigate potential sex differences in this context which we could not assess in view of a sample size of only eight males.

## 5 Conclusion

Modest weight loss in response to a 1.5-year combined lifestyle intervention (comprising a healthy normocaloric diet, exercise and CBT) was accompanied by changes in levels of long-term adiposity-associated hormones towards healthy levels, and no changes in levels of most short-term regulators of energy homeostasis. The clinical impact of alterations in levels of appetite-regulating hormones on long-term weight loss maintenance during modest weight loss remains questionable. Especially changes in levels of long-term adiposity-related hormones seem to rather follow weight loss instead of governing it. Future studies should investigate the potential association of (changes in) FGF21 and adiponectin levels with subsequent weight gain in a larger sample, ideally including measures of adipocyte distribution and health.

## Data Availability

The original contributions presented in the study are included in the article/supplementary materials, further inquiries can be directed to the corresponding author.
