# Supplementary material for: Levels of hormones regulating appetite and energy homeostasis in response to a 1.5-Year combined lifestyle intervention for obesity
Source: Front Physiol. 2023 Feb 20;14:1010858. doi: 10.3389/fphys.2023.1010858 (PMC9986487; doi:10.3389/fphys.2023.1010858)
Supplement: Supplementary file 1 [file DataSheet1.docx]

Supplementary Material

**Supplementary Table S1:** Associations of Long-term Changes in Hormone Levels (T0-T2) and Long-term Changes in Anthropometrics (T0-T2)

|  |  | **T0-T2 %∆** | | | | | | | | |
| --- | --- | --- | --- | --- | --- | --- | --- | --- | --- | --- |
|  |  | Leptin | Insulin | HMW Adiponectin | PYY | CCK | FGF21 | GIP | PP | AgRP |
|  |  | β  (95% CI) | β  (95% CI) | β  (95% CI) | β  (95% CI) | β  (95% CI) | β  (95% CI) | β  (95% CI) | β  (95% CI) | β  (95% CI) |
|  | **Uncorrected models ^a)^** | | | | | | | | | |
| T0-T2 %∆ | BMI | **2.577 *** ^#^ (1.581; 3.572)** | **4.117 *** ^#^ (2.221; 6.014)** | **-1.315 ** (-2.285; -0.345)** | -0.540  (-3.552; 2.473) | 1.319  (-0.811; 3.448) | 3.080  (-0.334; 6.495) | 1.111  (-2.162; 4.385) | 0.532  (-2.422; 3.485) | -0.894  (-2.190; 0.401) |
|  | WC | **1.452 ** (0.419; 2.484)** | **2.721 ** ^#^ (0.871; 4.572)** | -0.720  (-1.611; 0.171) | -0.575  (-3.160; 2.011) | 0.847  (-1.017; 2.712) | 0.723  (-2.334; 3.781) | -0.107  (-2.964; 2.750) | 0.320  (-2.243; 2.883) | **-1.243 * (-2.307; -0.180)** |
|  | **Models corrected for sex and age ^b)^** | | | | | | | | | |
| T0-T2 %∆ | BMI | **2.764 *** ^#^ (1.701; 3.826)** | **4.758 *** ^#^ (2.858; 6.658)** | **-1.635 ** ^#^ (-2.574; -0.697)** | -0.081  (-3.235; 3.074) | 0.700  (-1.502; 2.901) | 3.046  (-0.463; 6.556) | 1.992  (-1.422; 5.407) | 0.142  (-3.005; 3.288) | -1.208 (-2.553; 0.137) |
|  | WC | **1.529 ** (0.411; 2.647)** | **3.214 ** ^#^ (1.306; 5.122)** | -0.845  (-1.748; 0.057) | -0.021  (-2.747; 2.706) | 0.249  (-1.669; 2.166) | 0.842  (-2.314; 3.998) | 0.411  (-2.604; 3.426) | -0.142  (-2.869; 2.585) | **-1.535 ** (-2.629; -0.440)** |
| 95% CI = 95% confidence interval. ^a)^ Univariable linear regressions with T0-T2%∆ in BMI, or respectively WC, as predictor and T0-T2 %∆ in hormone levels as outcome. ^a)^ Multiple linear regressions, corrected for sex and age as potential confounders. *** significance at p< .001, ** significance at p<.01, * significance at p<.05. ^#^ significant after correction for multiple testing, p < .0055. N = 37 - 39 | | | | | | | | | | |
|  | | | | | | | | | | |

*Associations of Long-term Changes in Hormone Levels (T0-T2) and Long-term Changes in Anthropometrics (T0-T2)*

The observation that long-term T0-T2 decreases in BMI and WC correlated with long-term T0-T2 normalizations of adiposity-associated hormones (i.e. decreased leptin and insulin as well as increased HMW adiponectin) is in line with previous findings (Soni, Conroy, Mackey & Kuller, Menopause, 2011). In addition, greater T0-T2 decreases in WC tended to be associated with greater T0-T2 increases in orexigenic AgRP, suggesting that more extensive depletion of fat stores may have led to more orexigenic signaling in line with a hypothetical starvation response.

**Supplementary Figure S2:** Changes in Levels of Hormones Regulating Appetite and Energy Homeostasis in Response to a Combined Lifestyle Intervention from Baseline (T0) to 10 Weeks (T1) and 1.5 Years of Treatment (T2) ***in Women*.** A) Changes in levels of long-term adiposity-related hormones, including decreases in leptin and insulin levels towards normal levels. Dashed lines depict the upper limit of the reference range for leptin and insulin for healthy populations (33.9 ng/ml; ≤ 100 pmol/l respectively) as used for clinical diagnostics in the Erasmus MC Rotterdam. N=29-31. Data are expressed as mean +/- SEM (leptin) or median (IQR) (insulin, HMW adiponectin), depending on normal distribution. HMW Adiponectin = High-molecular-weight adiponectin**.** B) No change in levels of most short-term regulators of energy balance at the end of CLI. PYY = Peptide tyrosine-tyrosine, CCK = Cholecystokinin, FGF21 = Fibroblast growth factor 21, AgRP = Agouti-related protein, GIP = Gastric-inhibitory polypeptide, PP = Pancreatic polypeptide. Data are expressed as mean +/- SEM (PYY) or median (IQR) (PP, AgRP, GIP, PYY, CCK, FGF-21), depending on normal distribution * p < .05, *** p < .001 after repeated-measures ANOVA with Bonferroni-corrected post-hoc tests or Friedman’s test with Bonferroni-corrected post-hoc tests in case of non-normality. N=30-31.

A)

B)

Table S3: Associations Between Initial (T0-T1) % Changes in Hormone Levels and Subsequent (T1-T2) % Changes in Anthropometrics

|  |  |  |  | **Model 3 Adjusted for age, sex and T0-T1 % ∆ BMI / WC** | | |
| --- | --- | --- | --- | --- | --- | --- |
|  | ***N*** |  |  | ***β (95% CI)*** | ***Standardized β*** | ***p*** |
| Dependent variable:  T1-T2 ∆ % **BMI** |  |  |  |  |  |  |
| T0-T1 ∆ % Leptin  T0-T1 ∆ % Insulin  T0-T1 ∆ % HMW Adiponectin  T0-T1 ∆ % GIP  T0-T1 ∆ % PP  T0-T1 ∆ % PYY  T0-T1 ∆ % CCK  T0-T1 ∆ % FGF21  T0-T1 ∆ % AgRP | 39  39  39  38  38  38  39  38  38 |  |  | 0.006 (-0.105; 0.117)  -0.061 (-0.129; 0.006) **0.124 (0.009; 0.239)** 0.000 (-0.028; 0.029) -0.037 (-0.097; 0.022) -0.035 (-0.071; 0.002)  0.012 (-0.068; 0.093) **-0.021 (-0.041; -0.001)** -0.022 (-0.053; 0.097) | .027 -.316 **.374** .003 -.215 -.329 .057 -.**349** .110 | .908 .073 **.036*** .984 .210 .064 .756 .**042*** .560 |
| Dependent variable:  T1-T2 ∆ % **WC** |  |  |  |  |  |  |
| T0-T1 ∆ % Leptin  T0-T1 ∆ % Insulin  T0-T1 ∆ % HMW Adiponectin  T0-T1 ∆ % GIP  T0-T1 ∆ % PP  T0-T1 ∆ % PYY  T0-T1 ∆ % CCK  T0-T1 ∆ % FGF21  T0-T1 ∆ % AgRP | 39  39  39  38  38  38  39  38  38 |  |  | -0.019 (-0.125; 0.086) -0.024 (-0.099; 0.052) 0.097 (-0.029; 0.224) -0.015 (-0.045; 0.015) -0.024 (-0.087; 0.039) -0.020 (-0.062; 0.021) -0.018 (-0.115; 0.080) -0.013 (-0.036; 0.010) 0.006 (-0.077; 0.088) | -.072 -.106 .258 -.173 -.127 -.170 -.072 -.193 .025 | .709 .529 .127 .313 .437 .325 .711 .256 .890 |
| *CI = confidence interval, BMI = body-mass-index, WC = waist circumference, HMW adiponectin = high-molecular-weight adiponectin, GIP = gastric inhibitory polypeptide, PP = pancreatic polypeptide, PYY = peptide tyrosine-tyrosine, CCK = cholecystokinin, FGF21 = fibroblast growth factor 21, AgRP = agouti-related protein. Depicted are results of multiple linear regressions with T0-T1 % hormone changes as independent variables predicting T1-T2 % change in BMI/WC; corrected for age and sex as well as T0-T1 % changes in BMI (when dependent variable was T1-T2 % change BMI) or T0-T1 % change WC (when dependent variable was T1-T2 % changes in WC). * indicates p < .05. None of the association was significant at the Bonferroni-corrected significance level α = 0.005. 95% CI = 95% Confidence interval.* | | | | | | |

***Supplementary Table S4:*** ***Associations Between Initial (T0-T1) % Changes of Hormone Levels and Subsequent (T1-T2) % Changes in Anthropometrics in Women.***

|  |  |  | **Model 1 Unadjusted ^a^**^)^ | | |  | **Model 2 Adjusted for age ^b)^** | | |  | **Model 3 Adjusted for age and T0-T1 % ∆ BMI / WC ^c)^** | | |
| --- | --- | --- | --- | --- | --- | --- | --- | --- | --- | --- | --- | --- | --- |
|  | ***N*** |  | ***β (95% CI)*** | ***Standardized β*** | ***p*** |  | ***β (95% CI)*** | ***Standardized β*** | ***p*** |  | ***β (95% CI)*** | ***Standardized β*** | ***p*** |
| Dependent variable:  T1-T2 ∆ % **BMI** |  |  |  |  |  |  |  |  |  |  |  |  |  |
|  |  |  |  |  |  |  |  |  |  |  |  |  |  |
| T0-T1 ∆ % Leptin  T0-T1 ∆ % Insulin  T0-T1 ∆ % HMW Adiponectin  T0-T1 ∆ % GIP  T0-T1 ∆ % PP  T0-T1 ∆ % PYY  T0-T1 ∆ % CCK  T0-T1 ∆ % FGF-21  T0-T1 ∆ % AgRP | 31  31  31  30  30  30  31  30  30 |  | 0.003 (-0.098; 0.103) **-0.086 (-0.166; -0.005)** 0.127 (-0.007; 0.260) -0.008 (-0.040; 0.023) -0.042 (-0.115; 0.030) -0.037 (-0.077; 0.004) 0.030 (-0.060; 0.121) **-0.022 (-0.044; -0.001)** 0.063 (-0.038; 0.163) | .010  -.**374**  **.**339  -.102  -.219  -.333  .127  -.**369**  .234 | .957  .**038***  .062  .593  .244  .072  .497  **.045***  .213 |  | 0.003 (-0.096; 0.103)  -0.080 (-0.161; 0.001) 0.121 (-0.011; 0.254) -0.002 (-0.036; 0.032) -0.038 (-0.111; 0.035) -0.035 (-0.075; 0.006)  0.018 (-0.075; 0.110) **-0.022 (-0.043; 0.000)** 0.045 (-0.064; 0.155) | .013 -.351 .325 -.024 -.196 -.312 .074 **-.362** .170 | .945 .051 .072 .905 .299 .091 .698 .**047*** .401 |  | -0.011 (-0.152; 0.130)  **-0.089 (-0.174; -0.005)** **0.142 (0.001; 0.283)** -0.002 (-0.036; 0.032) -0.042 (-0.119; 0.034) -0.037 (-0.079; 0.005)  0.016 (-0.079; 0.112) -0.022 (-0.044; 0.000) 0.045 (-0.070; 0.159) | -.041 **-.389** **.380** -.023 -.220 -.333 .068 -.362 .167 | .875 .**039*** **.048*** .909 .265 .081 .730 .053 .430 |
| Dependent variable:  T1-T2 ∆ % **WC** |  |  |  |  |  |  |  |  |  |  |  |  |  |
| T0-T1 ∆ % Leptin  T0-T1 ∆ % Insulin  T0-T1 ∆ % HMW Adiponectin  T0-T1 ∆ % GIP  T0-T1 ∆ % PP  T0-T1 ∆ % PYY  T0-T1 ∆ % CCK  T0-T1 ∆ % FGF-21  T0-T1 ∆ % AgRP | 31  31  31  30  30  30  31  30  30 |  | -0.053 (-0.164; 0.058) -0.041 (-0.137; 0.056) 0.105 (-0.049; 0.260) -0.019 (-0.052; 0.014) -0.045 (-0.123; 0.033) -0.022 (-0.069; 0.026) -0.035 (-0.137; 0.066) -0.013 (-0.038; 0.013) 0.022 (-0.094; 0.138) | -.177  -.158  .251  -.220  -.219  -.175  -.132  -.186  .073 | .340  .395  .174  .243  .245  .355  .480  .324  .703 |  | -0.052 (-0.164; 0.060) -0.037 (-0.135; 0.062) 0.101 (-0.055; 0.257) -0.019 (-0.055; 0.017) -0.043 (-0.123; 0.036) -0.020 (-0.068; 0.028) -0.048 (-0.152; 0.056) -0.012 (-0.038; 0.014) 0.006 (-0.120; 0.132) | -.176 -.142 .241 -.214 -.211 -.162 -.179 -.182 .020 | .347 .452 .194 .295 .273 .399 .351 .339 .923 |  | -0.028 (-0.163; 0.106) -0.028 (-0.128; 0.073) 0.097 (-0.060; -0.253) -0.020 (-0.056; 0.016) -0.039 (-0.120; 0.041) -0.022 (-0.070; 0.026) -0.029 (-0.147; 0.089) -0.013 (-0.039; 0.013) 0.015 (-0.112; 0.143) | -.095 -.108 .230 -.232 -.192 -.174 -.109 -.198 .052 | .670 .575 .215 .257 .321 .364 .615 .298 .805 |
| *^a)^ Univariable linear regressions were used with T0-T1 % changes in hormone levels as predictor and T0-T2 % changes in BMI / WC as outcomes.* ***^b)^*** *Adjustment was performed using multiple linear regression.*  ***^c)^*** *Additional adjustment for T0-T1 % changes in BMI (when dependent variable T1-T2 % change BMI) or T0-T1 % change WC (when dependent variable T1-T2 % changes in WC). * indicates p < .05. None of the association was significant at the Bonferroni-corrected significance level α = 0.005. 95% CI = 95% Confidence interval.* | | | | | | | | | | | | | |

Supplementary Figure S5: Association of % Changes in HMW Adiponectin, Insulin, PYY and FGF21 Levels During Initial Weight Loss (T0-T1) and Subsequent Weight Gain (T1-T2). After removing the extreme values (red circles), the following associations were no longer apparent: HMW adiponectin [unadjusted analysis: β = 0.040 (-0.076; 0.157 95 % CI), p = .490; adjusted analysis (sex, age): β = 0.049 (-0.073; 0.170 95 % CI), p=.421]; and FGF-21 [unadjusted analysis: β = -0.009 (-0.029; 0.011 95 % CI), p = .367; adjusted analysis (sex, age): β = -0.008 (-0.029; 0.013 95 % CI), p = .422]. Data is expressed as individual dots representing individual patients with regression line and 95% CI (unadjusted analysis).

|  |  | **T0-T1 %∆** | | | | | | | | |
| --- | --- | --- | --- | --- | --- | --- | --- | --- | --- | --- |
|  |  | Leptin | Insulin | HMW Adiponectin | PYY | CCK | FGF21 | GIP | PP | AgRP |
|  |  | β  (95% CI) | β  (95% CI) | β  (95% CI) | β  (95% CI) | β  (95% CI) | β  (95% CI) | β  (95% CI) | β  (95% CI) | β  (95% CI) |
|  |  | | | | | | | | | |
|  | **Uncorrected models ^a)^** | | | | | | | | | |
| T0-T1 %∆ | BMI | **3.826 ***  (2.366; 5.287)** | 1.795  (-0.497; 4.087**)** | -0.947  (-2.301; 0.406) | 2.094  (-2.323; 6.510) | 1.439  (-0.617; 3.494) | -2.483  (-10.361; 5.395) | -0.079  (-6.063; 5.905) | 1.788  (-0.875; 4.451) | 1.391  (-0.954; 3.737) |
|  | WC | **1.833 ** (0.817; -2.848)** | 0.668  (-0.763; 2.099) | -0.160  (-1.008; 0.688) | -0.552  (-3.258; 2.154) | **1.741 ** ^#^ (0.588; 2.894)** | -1.238  (-6.024; 3.549) | -0.617  (-4.192; 2.958) | 0.349 (-1.281; 1.979) | 0.573 (-0.864; 2.011) |
|  |  | | | | | | | | | |
|  | **Models corrected for sex and age ^b)^** | | | | | | | | | |
| T0-T1 %∆ | BMI | **4.069 *** ^#^ (2.607; 5.532)** | 2.172  (-0.127; 4.471) | -1.105  (-2.428; 0.218) | 2.450  (-1.908; 6.807) | 1.025  (-0.984; 3.035) | -2.478  (-10.419; 5.462) | 0.846  (-5.108; 6.800) | 1.792  (-0.990; 4.574) | 0.980  (-1.258; 3.218) |
|  | WC | **1.920 ** ^#^ (0.889; 2.951)** | 0.830  (-0.604; 2.264) | -0.180  (-1.010; 0.651) | -0.297  (-2.976; 2.383) | **1.556 ** (0.443; 2.669)** | -1.014  (-5.825; 3.796) | -0.147  (-3.703; 3.409) | 0.328  (-1.369; 2.025) | 0.301  (-1.062; 1.665) |
| 95% CI = 95% confidence interval. ^a)^ Univariable linear regressions with T0-T1%∆ in BMI, or respectively WC, as predictor and T0-T1%∆ in hormone levels as outcome. ^a)^ Multiple linear regressions, corrected for sex and age as potential confounders. *** significance at p< .001, ** significance at p<.01, * significance at p<.05, ^#^ significant after correction for multiple testing, p<.0055. N = 37-39. | | | | | | | | | | |

**Supplementary Table S6:** Associations of Short-term Changes in Hormone Levels (T0-T1) and Short-term Changes in Anthropometrics (T0-T1)

*Associations of Short-term Changes in Hormone Levels (T0-T1) and Short-term Changes in Anthropometrics (T0-T1)*

The observation that long-term T0-T1 decreases in BMI and WC correlated with long-term T0-T1 decreases of leptin levels and, in trend, normalizations of insulin and HMW adiponectin, is partially in line with previous finidngs (Soni, Conroy, Mackey & Kuller, Menopause, 2011). In addition, greater T0-T1 decreases in WC tended to be associated with greater T0-T1 decreases in anorexigenic CCK, suggesting that more extensive depletion of fat stores may have led to more orexigenic signaling in line with a hypothetical starvation response.

**Supplementary Table S7:** Spaghetti Plots Showing Significant Changes in Levels of Hormones Regulating Appetite and Energy Homeostasis in Response to a Combined Lifestyle Intervention from Baseline (T0) to 10 Weeks (T1) and 1.5 Years of Treatment (T2)**.** HMW Adiponectin = High-molecular-weight adiponectin**,** AgRP = Agouti-related protein, GIP = Gastric-inhibitory polypeptide, PP = Pancreatic polypeptide. * p < .05, ** p < .01, *** p < .001 after repeated-measures ANOVA with Bonferroni-corrected post-hoc tests or Friedman’s test with Bonferroni-corrected post-hoc tests in case of non-normality. N=37-39.
